# Rectal endoscopic full-thickness resection of peritoneal loose body

**DOI:** 10.1055/a-2371-1322

**Published:** 2024-08-07

**Authors:** Minyuan Yang, Jinfeng Ren, Zhengying Yang, Xihui Yuan, Tianyu Liu

**Affiliations:** 1 529706 Unit 4, Gastroenterology Center, Suining Central Hospital, Suining, China; 2 529706 Endoscopy, Suining Central Hospital, Suining, China

A 36-year-old woman presented with recurrent lower abdominal pain for 6 months. Abdominal contrast-enhanced computed tomography (CT) showed a 1.2-mm enhancing lesion in the upper rectum ( Fig. 1 ). Intestinal endoscopy revealed a 1.5 × 1.2-cm rectal mass located 15 cm from the anal dentate line ( Fig. 2 ). Mucosal biopsy indicated mild chronic inflammation. Endoscopic ultrasonography revealed a 2.2-cm submucosal lesion with an intact mucosal layer ( Fig. 3 ). After obtaining consent from the patient, submucosal tumor endoscopic resection was planned, but formation of the liquid pad by injection proved difficult. The margins of the lesion on the mucosal surface were marked using electrocoagulation ( Fig. 4 ). After obtaining a second consent, rectal endoscopic full-thickness resection was performed ( Video 1 ). A balloon catheter was placed in the sigmoid colon to block fecal matter. An endoscopic mucotomy knife (KD-650Q; Micro-Tech Co., Ltd., Nanjing, China) was used to incise the marked mucosa until an orifice formed. Then, an IT knife (KD-650L; Micro-Tech Co., Ltd.) was used to incise the full thickness around the lesion. After inspecting the outer intestinal wall fascia, an endoscopic electrified snare was used to dissect the lesion into two sections. The defect in the rectal wall was sutured using three three-armed clips (STA00002; Micro-Tech Co., Ltd.) and several titanium clips (ROCC-D-26–195-C; Micro-Tech Co., Ltd.). Histological examination of the biopsy specimen showed chronic submucosal inflammation with foam cell reaction and no abnormal cells at the horizontal and vertical margins. During the operation, no gas entered the abdominal cavity, all abdominopelvic cavity organs remained unaffected, and there was minimal bleeding. Follow-up after 6 weeks showed no residual mass on contrast-enhanced CT, with only scars and two clips at the junction site observed.

**Fig. 1:**
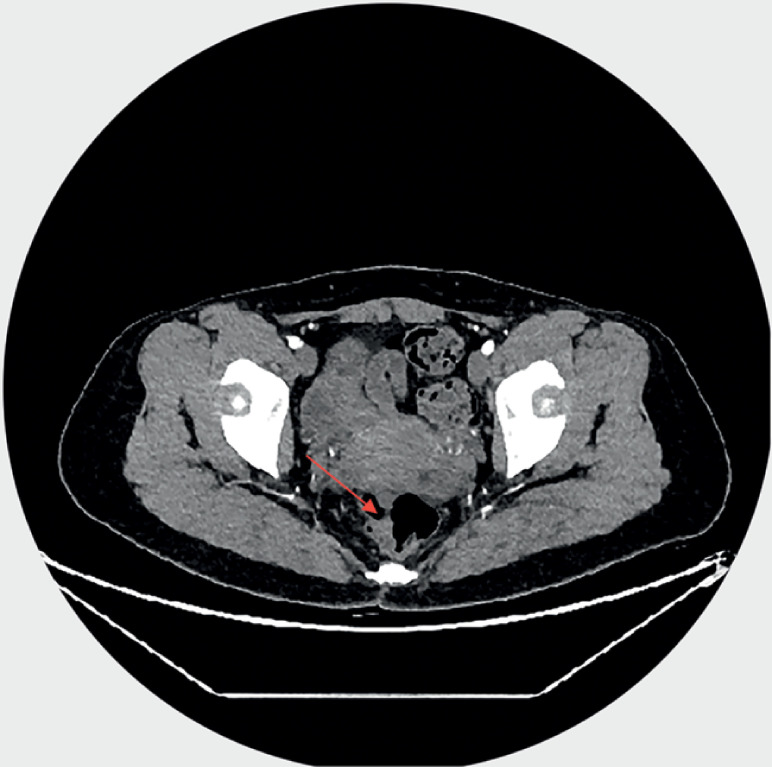
Preoperative abdominal contrast-enhanced computed tomography showing a lesion (red arrow) in the upper rectum.

**Fig. 2:**
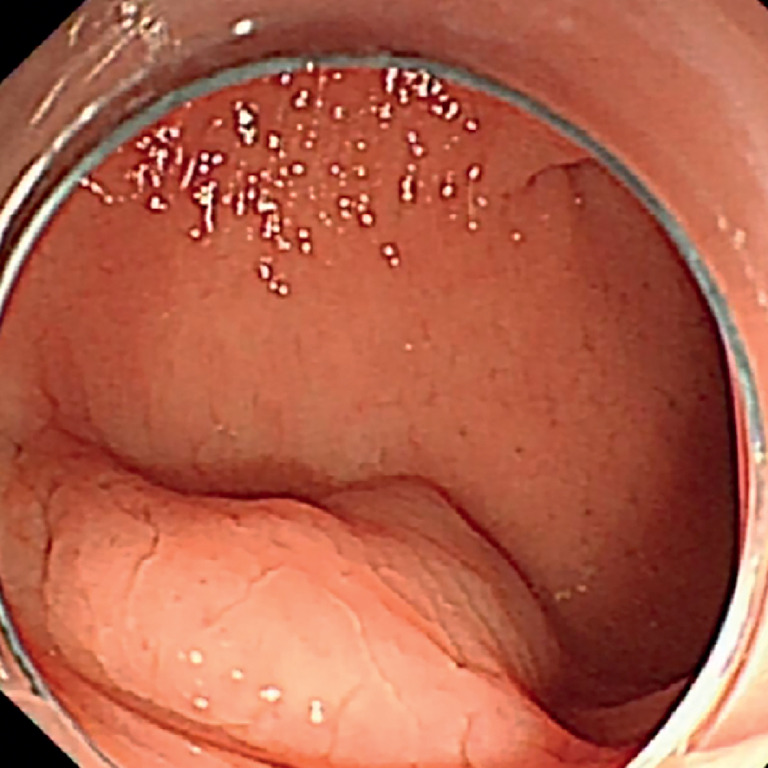
Preoperative intestinal endoscopy showing a rectal mass.

**Fig. 3:**
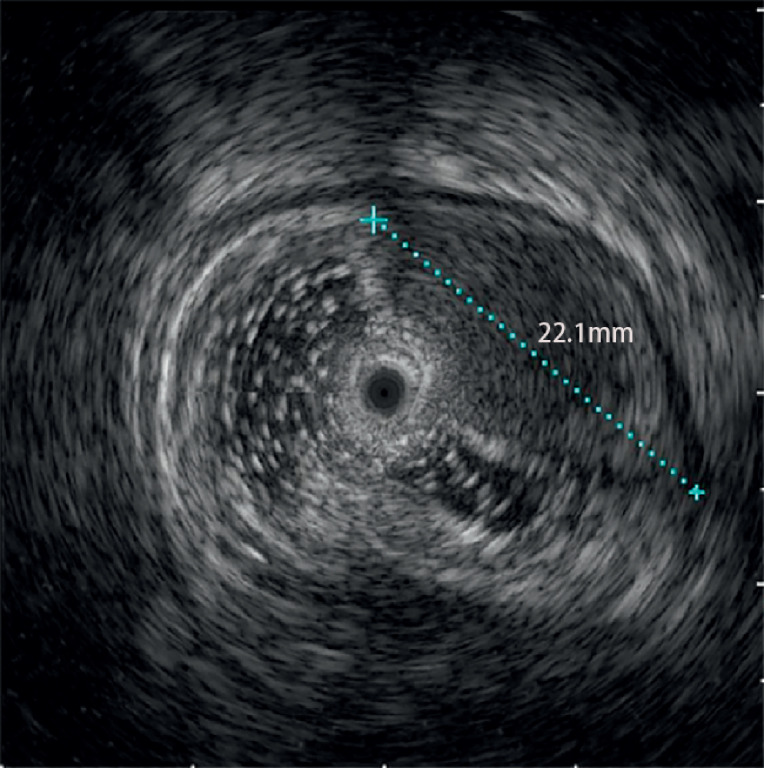
Endoscopic ultrasonography showing a 2.2-cm submucosal lesion.

**Fig. 4:**
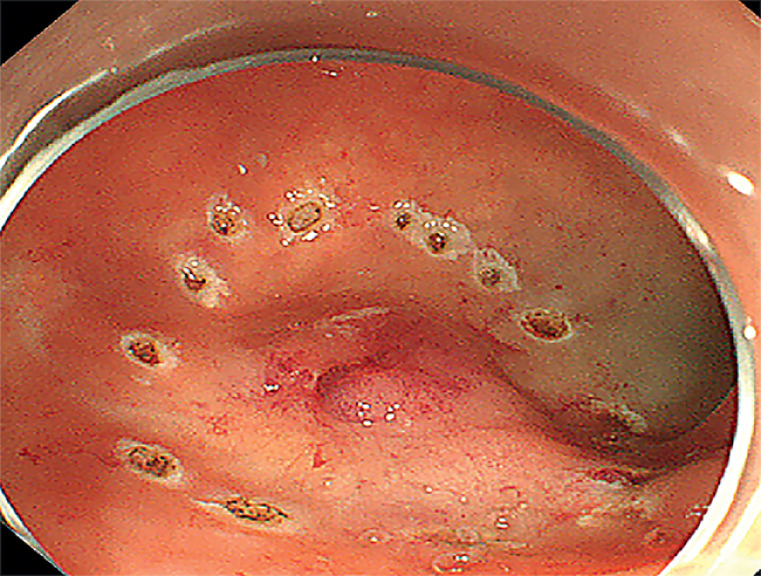
After failing to create a liquid pad by injecting epinephrine and methylene blue, electrocoagulation was used to delineate the margins of the lesion.

Rectal endoscopic full-thickness resection of a peritoneal loose body in a 36-year-old woman.Video 1

The postoperative biopsy revealed foam cells, consistent with the characteristics of peritoneal loose bodies 1 . This is the first reported case of endoscopic resection of a peritoneal loose body. The endoscopic procedure demonstrated efficiency and safety in this case. However, large-scale clinical studies are needed in the future.

Endoscopy_UCTN_Code_TTT_1AQ_2AH

## Notes

Endoscopy E-Videos https://eref.thieme.de/e-videos
*E-Videos* is an open access online section of the journal *Endoscopy* , reporting on interesting cases and new techniques in gastroenterological endoscopy. All papers include a high-quality video and are published with a Creative Commons CC-BY license. Endoscopy E-Videos qualify for HINARI discounts and waivers and eligibility is automatically checked during the submission process. We grant 100% waivers to articles whose corresponding authors are based in Group A countries and 50% waivers to those who are based in Group B countries as classified by Research4Life (see: https://www.research4life.org/access/eligibility/ ). This section has its own submission website at https://mc.manuscriptcentral.com/e-videos .
